# Mindfulness as a Weight Loss Treatment for Veterans

**DOI:** 10.3389/fnut.2016.00030

**Published:** 2016-08-15

**Authors:** Michael V. Stanton, Justin Matsuura, Jennifer Kaci Fairchild, Jessica A. Lohnberg, Peter J. Bayley

**Affiliations:** ^1^Stanford University, Stanford, CA, USA; ^2^Veterans Affairs Palo Alto Health Care System, Palo Alto, CA, USA; ^3^US Army Health Clinic Schofield Barracks Brain Injury Clinic, Schofield Barracks, HI, USA

**Keywords:** veterans, veterans health, mindfulness, obesity, weight loss

## Abstract

Despite substantial evidence for their effectiveness in treating disordered eating and obesity, mindfulness-based treatments have not been broadly implemented among Veterans. A number of reviews have reported mindfulness to be beneficial in promoting healthy eating behaviors and weight loss among non-Veteran samples. We discuss this approach in the context of the Veterans Affairs system, the largest integrated healthcare provider in the U.S. and in the context of Veterans, among whom obesity is at epidemic proportions. In this article, we discuss what is known about treating obesity using a mindfulness approach, mindfulness interventions for Veterans, a new pilot mindfulness-based weight loss program designed for Veterans, and future directions for this type of obesity treatment in Veterans. We conclude that this population may be uniquely poised to benefit from mindfulness-based treatments.

## Introduction

Mindfulness has been defined as a form of mental training that aims to foster “the awareness that emerges through paying attention on purpose, in the present moment, and non-judgmentally to the unfolding of experience moment by moment” [Ref. (1), p.145–146]. As implied by the broad definition, the term “mindfulness” has been used to describe a diverse range of practices that emphasize focused attention on present moment experience without judgment (1). Mindfulness can involve physical, mental, and emotional experiences (1–3). Research has shown that mindfulness is a skill that can be taught, acquired, and practiced (4). Although physical and mental health consequences are not emphasized in its use as a core Buddhist teaching, its practice is becoming increasingly popular, partly for its purported benefits for general health and psychological well-being.

In this perspective, we focus on mindfulness as a treatment for obesity in Veterans. As we will discuss below, a number of reviews have investigated mindfulness for various types of obesity-related eating behaviors, and these studies have reported mindfulness to be of benefit in promoting weight loss among non-Veterans. We discuss this approach in the context of treating the Veteran population, since the Veterans Health Administration is the largest integrated healthcare provider in the U.S. consisting of more than 1,000 medical care sites and treating more than 8.3 million Veterans annually (5). Obesity in Veteran populations is at epidemic proportions; 78% of Veterans are obese vs. 69% of the U.S. general population (6, 7). Over 10 years, Veterans with class III obesity cost the VA $8.9 billion dollars more than normal weight Veterans, and these costs are projected to continue to soar in the future (6, 8). Moreover, the health effects of obesity can be even greater among Veterans, since obesity often co-occurs with other medical and psychological challenges associated with factors such as combat exposure, deployments, and unexpected relocations (9).

The purpose of this paper is multipronged, as we will discuss what is known about treating obesity using a mindfulness approach, mindfulness studies for Veterans, and a new pilot weight loss program designed for Veterans using mindfulness techniques. Finally, we consider future issues regarding the use of mindfulness in treating obesity among Veterans.

Mindfulness-based treatments often guide participants to perform particular practices, such as mindful walking, meditation, or eating (see Table 1 for mindfulness definitions). Some clinicians and researchers, including the authors of this article, also expand the scope of mindfulness-based practices to include those practices that do not explicitly teach mindfulness exercises. Dubbed “acceptance and mindfulness procedures” by Hayes or “acceptance-based” practices by Forman and others, these treatments focus on the principle of “non-judgment” and guide participants to incorporate intentional attention, present moment focus, and a non-judgmental attitude into their daily life without emphasizing mindfulness exercises [e.g., mindful walking or sitting; (10), p. 101; (11)]. Commonly practiced mindfulness-based and acceptance-based therapies include acceptance and commitment therapy [ACT; (12)], dialectical behavior therapy [DBT; (13)], and mindfulness-based cognitive therapy [MBCT; (14)].

**Table 1:** **Description of common mindfulness techniques**.

Mindfulness techniques		
Technique	Description	Example of instruction
Mindful eating exercise	Eating with conscious control over the process of eating and awareness and non-judgment of sensory stimuli	Take a raisin in … your hand … imagine that you have just dropped in from Mars and have never seen an object like this before … now, prepare to chew the raisin, noticing how and where it needs to be for chewing
Mindful walking exercise	Walking with conscious control over the process of walking and awareness of proprioceptive and other stimuli without judgment	Bring the focus of your awareness to the bottom of your feet, getting a direct sense of the physical sensations of the contact of the feet with the ground and of the weight of your body transmitted through your legs and feet to the ground
Mindful breathing exercise	Breathing with awareness of the associated, interoceptive sensations and without judgment	Notice expansion and contractions of your lungs … the subtle movements in your chest and shoulders … the air passing through your nostrils
Body scan exercise	Paying attention to interoceptive cues in successive parts of the body without judgment	Notice any and all sensations in your left foot, whatever they may be. Perhaps, sensations of contact with your shoes, warmth, tingling, or pain … now shift your attention to your lower left leg
Meditation	Intentional focus on one’s breath, a word, or other object, often with an emphasis on sustaining attention and then returning attention in the event of mind-wandering	[Mind-Wandering] is a natural tendency of the mind … whenever you notice that your attention has drifted … gently bring it back to [the breath, your contact with the floor, etc.]
Present-moment focus	Concentration on what is happening in the present moment	Notice what you are experiencing in the present moment, when you notice you are thinking about the future or past, return your attention to the present
Mindfulness of thoughts	Noticing the presence of thoughts without judging them as good or bad and without any effort to change them	Bring a gentle openness and interest to your [troublesome thought] with awareness and a non-judgmental attitude

General studies on the effectiveness of mindfulness for various indicators have reported positive results. General outcomes of mindfulness practice include self-control, objectivity, affect tolerance, enhanced flexibility, equanimity, improved concentration and mental clarity, emotional intelligence, and the ability to relate to others and one’s self with kindness, acceptance, and compassion [for a review, see Ref. (3)]. A large comprehensive meta-analysis of 209 mindfulness-based therapy studies reported moderate to large effects in pre- and post-studies and across other waitlist controlled studies, especially for reducing anxiety, depression, and stress (15).

Specific studies on the effectiveness of mindfulness-based interventions for disordered eating and weight loss have also reported positive results among non-Veteran samples. These interventions have been associated with improvements in emotional eating and binge eating behaviors (16, 17), as well as significant weight loss in 13 out of 19 studies in a recent review (18).

Although cognitive behavioral therapy (CBT) has been understood as the gold standard psychotherapeutic intervention in obesity treatment (19), these treatments produce relatively low adherence rates to diet and physical activity recommendations, and CBT participants lose far less weight than those in a controlled environment (20, 21). In addition, most participants regain most, if not all, of their weight within 5 years (22–24). There is emerging evidence that mindfulness interventions intervene at the points where CBT fails: making and/or maintaining recommended changes in dietary and physical activity (11).

Mindfulness-based skills could be useful in weight management for a number of reasons. Being aware of one’s intake and expenditure of calories is critical to weight loss. Contrary to mindfulness, “mindless eating,” which has been defined as “ways in which environmental factors trigger eating without conscious processing,” has been associated with greater food consumption in a number of studies [(25), p. 17; (26–28)]. Thus, it has been suggested that self-regulation through monitoring one’s diet and physical activity is essential for successful weight management.

Moreover, weight management is often associated with uncomfortable sensations and emotions (e.g., hunger, physical exertion, and sore muscles) that may be better tolerated and managed with mindfulness and acceptance-based techniques (11). As the authors of one review concluded, “individuals who exhibit greater mindfulness may be more resilient and better prepared to confront the challenges of weight management” (18).

## Mindfulness and Obesity in Veterans

Among all studies on the use of mindfulness in Veterans, we identified only one published study that used mindfulness to treat disordered eating or obesity. This study reported that participation in mindfulness training alone did not influence eating behaviors; the intervention was associated with small increases in weight and body mass index at 4-month follow-up (29). However, the intervention enhanced mindfulness skills and reduced depressive symptoms over time, with medium to large effect sizes [Cohen’s *d* = 0.69 (95% CI = 0.27–1.11) and 0.74 (95% CI = 1.16–0.33), respectively]. Moreover, improvement in mindfulness skills was significantly and negatively correlated with changes in emotional and uncontrolled eating over time.

Apart from the results of this single study, the effectiveness of mindfulness treatments for obesity among Veterans or men in general is unknown. Mindfulness-based techniques have been associated with weight loss primarily in younger, female samples but have not been tested in the Veteran population, which is primarily older and males. As described in a review article of 14 studies on mindfulness and eating behavior or obesity, the majority of participants in these studies are women of age 40–60 years old, whereas most Veterans are men with a median age of 64 years old (16, 30). Moreover, nearly three-quarters of a sample of 45,477 overweight and obese Veterans report emotional eating behaviors and binge eating behavior for which men were at a particularly high risk (31). Mindfulness has also been useful in reducing symptoms for a number of common comorbid conditions in Veterans including post-traumatic stress disorder (PTSD), anxiety, and depression, as described below.

An attractive feature of these treatments is that, by teaching Veterans mindfulness-based skills for weight loss, one might improve various other mental health conditions as well. Conversely, there is some evidence that treatments, which lead to improvement in other mental health conditions, may facilitate better weight loss outcomes among those with mental health issues. For example, previous work has suggested an association between worse mental illness symptomatology and weight gain (32, 33). And, lifestyle interventions that improve weight loss also improve mental health symptoms (34, 35). These associations suggest that mindfulness-based interventions might be an effective treatment for common mental illnesses and obesity, among Veterans.

The mindfulness literature among Veteran samples has primarily focused on its usefulness in treating conditions such as depression and PTSD, common comorbidities among Veterans. Regarding mindfulness research focusing on Veterans, 38 articles on PubMed included (the string “veteran* and mindfulness”) in the title and abstract of articles. Among these articles, the majority (*n* = 23) focused on PTSD. The effect of mindfulness on PTSD in Veterans has demonstrated promising results (36–40). For example, one review found positive effects on depression and PTSD in the majority of studies (41). As described earlier, a large meta-analysis of 209 mindfulness-based studies reported significant effects in reducing anxiety and depression (15). Depression, anxiety, and PTSD are also all conditions that are found more commonly in Veteran samples than in the general population (42, 43).

Taken together, preliminary results suggest that basic mindfulness training can be successful in reducing symptoms of depression, anxiety, and PTSD in Veterans, but additional evidence is required to address its effect on obesity. The only prior published study on disordered eating among Veterans (29) was limited by an intervention focused solely on mindfulness and stress reduction. We conjecture that, if this basic mindfulness training were combined with more specific instruction on nutrition, physical activity, and social behavior related to eating in Veterans, this more comprehensive intervention would be more effective in promoting weight loss and other healthy behaviors.

## Mindfulness Intervention Design

The VA system addresses obesity among Veterans through the MOVE! program (44). The actual dissemination of the program varies across the country, but the minimal requirements of the MOVE! program include “screening all Veterans for … obesity-related conditions, referring Veterans for whom weight management is appropriate …, conducting a multifactorial assessment, and providing support for weight self-management in … individual or group care” [(45), p. 2]. The weight loss group component is a common feature across sites for which 16 weekly sessions are described online, but fewer sessions are typically implemented, due to group leader and participant time limitations. Among these sessions, only 13 min of group session time and one 2-page handout are allotted to discussing mindful eating. No other mindfulness materials appear in the MOVE! program.

Based on the potential of a more comprehensive intervention, we designed a pilot mindfulness-based weight loss group for Veterans. This pilot study was designed to determine the feasibility of recruiting Veterans for such an intervention and deliver a manualized mindfulness-based intervention. As such, this study does not include a control group and thus cannot speak to the efficacy of mindfulness compared to other, non-mindfulness interventions. We expected that Veterans would be motivated to join for the goal of weight loss, but we did not know how they might react to the mindfulness techniques. Our intervention was created by psychologists and trainees at the VA Palo Alto Health Care System and based on Brownell’s book (46) entitled “LEARN Program for Weight Management 2000,” which compiles years of psychological and behavioral research on weight loss and Williams’ book (47) entitled, “The Mindful Way Through Depression: Freeing Yourself from Chronic Unhappiness,” which combines cognitive therapy and mindfulness training as described by Kabat-Zinn (48); (a) into a type of therapy known as MBCT.

Similar to MBCT, our intervention was derived from both CBT and mindfulness-based stress reduction (MBSR), the group practice on which much of the mindfulness literature above is based (49, 50). Furthermore, our intervention included some elements of traditional CBT, such as “behavior chain analysis,” which is a technique designed to help a person understand the function of a particular behavior by uncovering the many proximal and distal factors that led up to that behavior. The intervention excluded other elements of CBT such as “cognitive restructuring” (i.e., changing the content of a maladaptive thought) in favor of “mindfulness of thoughts” (see Table 1). The current intervention used imagery that better resembled the actual Veteran population and emphasized examples relevant to the Veterans’ lives. It was built upon other services offered by VA and was envisioned as an auxiliary therapy, in this respect.

The current intervention was designed as an 8-week protocol for a group of 10–15 male and female Veterans. Each session included a lesson related to weight loss, followed by mindfulness instruction, and a mindfulness exercise. The subject matter of initial classes (see Table 2) included basic nutrition and physical activity education; however, the focus of this intervention was CBT and mindfulness techniques for making effective behavior changes. CBT elements included a chain analysis of problematic health behaviors and recognizing automatic negative thoughts that interfere with healthy living. Classes also focused on social interaction, intending to improve communication among friends, family, and people they encountered in eating-related situations.

**Table 2:** **Mindfulness weight loss intervention class schedule**.

Class schedule
Week 1 – diet vs. lifestyle; recordkeeping; exercise Diet = temporary; lifestyle = life change Recordkeeping is key Every bit of physical activity is important Mindful eating exercise Week 2 – a balanced diet; high risk situations Calculating and defining calories Eating a balanced diet Being mindful of behavior triggers Mindful walking exercise Week 3 – barriers to weight loss; emotions/stress Discuss individual high risk situations Describe role of emotions in eating Stress reduction through “being present” Without judging (i.e., mindfulness practice) Focused breathing exercise Week 4 – overcoming barriers, identify life values Leading a value-guided life Strategies for mindful eating Tips for eating out Body scan exercise Week 5 – Thoughts/feelings/behaviour, exercise Connecting thoughts, emotions, behaviors Effective coping strategies Reasons for physical activity Group-chosen mindfulness exercise Week 6 – social support; communication training Recognizing automatic negative thoughts Importance of social support Mindfulness in daily life Group-chosen mindfulness exercise Week 7 – Behavior chain analysis Behavior chain analysis description Creating your own behavior chain Mindfulness/mindlessness and eating Group-chosen mindfulness exercise Week 8 – relapse prevention; review successes Review of favorite skills Review of mindfulness skills Relapse vs. setback Group-chosen mindfulness exercise

The group leader taught both general and eating-specific mindfulness skills. For example, certain lessons focused on eating mindfully (i.e., as opposed to “eating mindlessly”) or mindfully keeping track of physical activity; mindfulness exercises taught at the end of each group typically focused on cultivating general mindfulness skills (e.g., mindful walking and mindful breathing) that incorporated focused attention on the present moment with a non-judgmental attitude. The final four classes reviewed a previously taught mindfulness skill selected by the group members. The group leader also referenced research describing the putative usefulness of mindfulness in improving abilities thought to be related to weight loss [e.g., concentration, self-control, emotion regulation, and decreasing stress; (3, 51)].

Once participants acquired specific mindfulness skills, the instructor regularly reminded group members how to apply these techniques throughout the week, particularly in the context of situations that might lead to disordered eating or less physical activity. This procedure contrasts with the only other study published on mindfulness and eating in Veterans, in which no explicit connection was drawn between improvement in mindfulness skills and improvement in eating behavior or other emotion or stress-related factors associated with losing weight.

In addition to mindfulness exercises, the course emphasized mindfulness concepts in-class. For example, the instructor described the concept of “acceptance” through class topics such as “Leading a Value-Driven Life,” which emphasized the ability to tolerate discomfort or difficulty in certain parts of one’s life on the path toward fulfilling a higher goal in line with one’s values. In this way, an individual might need to tolerate the discomfort associated with passing by fast food restaurants in order to practice avoiding unhealthy food. This process would help him/her fulfill his/her goal of weight loss and promote their value of good health. These concepts reflect core ACT concepts (12). In addition to 1.5 h of weekly in-class instruction, the intervention required participants to monitor their daily physical activity and diet and practice mindfulness exercises for 30 min daily at home.

## Pilot Intervention Findings

The pilot intervention was implemented at the VA Palo Alto Health Care System (VA Palo Alto) in 2014. The VA Palo Alto is a Stanford University-affiliated teaching hospital that serves more than 85,000 enrolled Veterans and maintains one of the top three research programs in the VA system. The VA Palo Alto’s Behavioral Medicine Clinic treats Veterans’ medical problems using behavioral interventions and addresses coping with chronic illnesses. Sixteen Veterans were recruited from the VA Palo Alto’s MOVE! Weight Loss Program. In total, 11 participants [mean age: 67 years; 10M/1F; mean baseline weight (lbs.): 249.5] completed at least 4 out of the 8 sessions. The institutional review board of Stanford University approved data collection.

Since this intervention was part of a clinical program and not a formal research study, care was taken to minimize participant burden. Therefore, few psychosocial outcome measures were administered. Measures included the Beck Depression Inventory-II (52), an updated version of the Questionnaire on Eating and Weight Patterns-Revised (53), and a novel four-item scale designed to assess participants’ motivation and confidence in making weight-related behavioral changes. Weight was measured weekly at the beginning of every class using a digital scale.

Over time, we observed decreases in average participant weight. Exploratory analyses revealed a trend toward reducing weight over time (mean = 1.76 lbs. lost) among the 11 participants that completed at least 4 out of 8 total classes over the 8-week program. The relationship between class attendance and weight loss was non-significant (*p* > 0.05). No other psychosocial outcome measures were significant.

Informal feedback suggested that participants enjoyed the program. One Veteran stated, “Thank you for the class. The group was very good. I enjoyed sitting and listening. It made learning better.” Another Veteran noted, “I found joining with the group and attending the class very enjoyable and enlightening. You quickly relaxed in this very diverse group, and it brought us all together with … respect and understanding of each other. One of the highlights that I take away from your class was that of ‘mindfulness.’ I’ve used [it] in my daily life and it’s been extremely helpful in undertaking life’s challenges.” Other Veterans noted that they enjoyed learning about mindfulness, and how to practice these skills in their daily lives.

There were several limitations in this exploratory trial. Although participants seemed to enjoy the intervention and learn useful strategies for weight loss, only a trend toward weight loss was observed. Significant weight loss may require more time and practice. For example according to MBSR^1^ or MBCT materials^2^, programs typically consist of 8 weekly 2.5-h sessions and one “all-day” weekend class. This current trial consisted of only 8 weekly 90-minute sessions. Due to logistical limitations, no participant acceptability measures were administered. This intervention contained components known to be associated with weight loss (e.g., group interventions and elements of the LEARN protocol); therefore, it cannot be determined that mindfulness components specifically provided an added benefit. This trial was also limited by recruitment difficulties due to transportation difficulties, multiple medical appointments, and family commitments that limited their ability to complete the intervention. A more formal pilot trial with a non-mindfulness comparison group and a larger sample would provide more power and might test a greater number of hypotheses. A controlled comparison group is also needed in order to substantiate claims about the benefits of mindfulness compared to other, non-mindfulness-based interventions.

## Discussion

This perspective and pilot study suggests that mindfulness-based interventions may be useful to Veterans who struggle with obesity, particularly those for whom other strategies have been unsuccessful or those resistant to other weight loss interventions. Certain mental illnesses are more common in Veterans, and these illnesses also seem to benefit from mindfulness interventions. Conversely, it is possible that the treatment of other mental illnesses might also be associated with increased weight loss. These data provide preliminary evidence to support a new approach to weight loss for Veterans. In this pilot, Veterans stated that they enjoyed the program, and satisfaction was evidenced by the large percentage (83%) of Veterans who completed the intervention after attending at least two sessions.

## Future Directions

A number of factors might moderate the effect of mindfulness-based interventions on eating behaviors and obesity among Veterans. Based on findings from Forman et al. (54, 55), it is hypothesized that mindfulness skills that help manage emotions associated with food (e.g., tolerating discomfort around unhealthy foods without approaching them and uncomfortable social situations without triggering binges) will be instrumental to the success of future studies.

We continue to explore utilizing practical mindfulness exercises to strengthen skills (e.g., focused concentration, purposeful distraction, prioritizing, and following through with priorities) linked to weight loss. Like any skill, it is likely that more frequent practice of mindfulness exercises (e.g., particularly mindful eating) will provide larger effects on eating behavior and weight change. For example, repeated mindfulness practice is thought to improve executive function abilities associated with strengthened prefrontal lobe circuits (56) and might aid in making decisions regarding challenging food (i.e., avoiding unhealthy and approaching healthy food) and physical activity (i.e., avoiding sedentary and increasing exercise behavior) decisions (18). That said, in our study, we did not observe any relationship between weight loss and class attendance (e.g., “practice”). Thus, it remains to be determined whether this technique offers improved benefit to regular users who practice the technique frequently.

There is also some evidence for individual differences in response to mindfulness training, including differential responses due to temperament, personality, or genetic differences (57), suggesting that some individuals might benefit more from mindfulness practice than others. Finally, it is possible that similar results may have been observed with a non-mindfulness-based intervention; since we did not include a control intervention, we cannot determine whether the mindfulness components of our intervention were primarily responsible for the trends in weight loss or the satisfaction of group members. Future studies with a control condition are necessary to demonstrate the added benefit of mindfulness-based components.

Although not yet regularly used within the VA Health Care System, mindfulness-based interventions have the potential to contribute to various improved health behaviors including weight loss. Integrating these treatments into existing weight loss programs might be the most efficacious method of implementing this project system-wide.

## Author Contributions

Dr. MS is the primary author who wrote and edited much of the text. Dr. MS also conducted the pilot study in the publication and analyzed the data. Drs. JF and JL provided clinical supervision for Dr. MS throughout this project and edited this manuscript. Dr. JM spearheaded and designed the pilot group intervention and edited the manuscript. Dr. PB supervised the writing of the manuscript and wrote and edited the text.

## Conflict of Interest Statement

The authors declare that the research was conducted in the absence of any commercial or financial relationships that could be construed as a potential conflict of interest.

## Footnotes

^1^http://www.umassmed.edu/cfm/stress-reduction/mbsr-8-week/

^2^http://mbct.com/mbct-classes/
